# Biological characteristics, bioactive compounds, and antioxidant activities of off-season mulberry fruit

**DOI:** 10.3389/fpls.2022.1034013

**Published:** 2022-10-25

**Authors:** Peigang Liu, Yan Zhu, Jingjing Ye, Tianbao Lin, Zhiqiang Lv, Zilong Xu, Lushan Xu, Leyang Chen, Jia Wei

**Affiliations:** ^1^ Institute of Sericulture and Tea, Zhejiang Academy of Agricultural Sciences, Hangzhou, China; ^2^ Sericultural Research Institute, Sichuan Academy of Agricultural Sciences, Nanchong, China; ^3^ Economic Specialty Technology Extension Station, Jinhua Municipal Bureau of Agriculture and Rural Affairs, Jinhua, China

**Keywords:** biological characteristics, bioactive compounds, antioxidant activities, off-season, mulberry fruit

## Abstract

To understand the yield and quality of off-season mulberry fruits, which are cultivated in open fields from autumn, the biological characteristics, bioactive compounds, and antioxidant activities of them were analyzed. Compared with mulberry fruits in normal season, the fruit length, fruit diameter, single fruit weight, fruit yield per meter strip, and the fruits yield per 667 m^2^ are significantly lower. The moisture content and juice yield of off-season mulberry fruits are lower than the mulberry fruits in normal season; the pH and soluble solids are higher. The contents of mass fraction of crude protein, total sugar, reducing sugar, total acids, total anthocyanins, and total flavonoids decreased significantly in all batches of off-season mulberry fruits compared with those of normal season. Of off-season mulberry fruits, the contents of glucose, fructose and sucrose, expression, anthocyanin biosynthesis genes, and antioxidant capacity are significantly lower than those in normal season.

## Introduction

Mulberry (*Morus alba* L.) belongs to the Moraceae family and has been regarded as a crop solely of economic importance in sericulture. The mulberry varieties which have female or androgynous flowers can bear fruit, and the varieties with high fruit yield have been bred mainly for fruit production (Vijayan et al., 2018; Krishna et al., 2019). Consumption of mulberry fruit (*Fructus mori*) either raw or dried has a long history. Nowadays, the fruit are consumed in processed forms, such as jams, juice, beverages, syrups, and liquor (Sadia et al., 2014; Ma et al., 2020). In addition, mulberry fruit have been used as a traditional oriental medicine from ancient times. The fruit may have positive effects on human health by protecting against diseases of the liver, gall bladder, and heart (Zhang et al., 2018; Bhattacharjya et al., 2021).

Given their nutritional characteristics and medicinal value, the demand for mulberry fruit by consumers is ever increasing. The harvesting of fruit of most mulberry varieties is from April to May, and thus the annual fruit production period of mulberry is short (Keskin and Özkan, 2020). In addition, the fruit are fragile and perishable, and are difficult to transport and store, which severely limits the economic viability of planting mulberry for fruit production and its expansion to an industrial scale (Singhal et al., 2010). Off-season cultivation of plants in a favorable controlled growth environment (e.g., greenhouses) and related technology enable modification of the normal growing season. Such technology is mainly utilized for cultivation of vegetable, fruit, and flower crops to meet consumer’s demand in off-season growing periods and the potential for higher returns (Kandel et al., 2020; Ramasamy et al., 2021). With technological developments, off-season cultivation has been developed and applied for mulberry fruit production. This approach not only enriches the fruit market and provides consumers with greater choice, but also financially benefits growers through obtaining higher market prices.

Cultivation of commercial crops in the off season or throughout the year is predominantly achieved by using different types of greenhouses to maintain a controlled growth environment. Greenhouses are mainly used for production of mulberry fruit in early spring (Kim et al., 2012; Lee et al., 2015a; Ahn et al., 2021). Greenhouse construction and energy consumption for climate control greatly increase the production costs for off-season cultivation, even with limited development of greenhouses (Shen et al., 2018). In Zhejiang province of China, mulberry trees naturally fruit in spring but have been successfully induced to produce successive fruit crops in autumn by treatment with paclobutrazol and monocyandiamide. By adjustment of the treatment time of paclobutrazol and monocyandiamide, mulberry trees can fruit at different times in an open field. This cultivation technique enables fresh mulberry fruit production in the off-season, significantly increases the income of growers, and reduces the required production costs, especially the need for greenhouses.

To date, there were no studies have investigated the biological characteristics and nutritional qualities of off-season mulberry fruit produced in an open field. In the current work, the yield and quality of mulberry fruit produced in the off-season and the normal season were compared to gain an improved understanding of the practical application of off-season production of mulberry fruit. The results provide data to support the potential for off-season mulberry fruit harvesting in autumn and lay a foundation for expansion of off-season mulberry fruit production.

## Materials and methods

### Plant material and fruit collection

Three years old mulberry ‘Da10’ was planted at the Junxin family farm, Huashui Town, Dongyang City, China (29°15′ N, 120°08′ E) in 2018. The grafting rootstock for ‘Da10’ was ‘Guiyou 12’, and planted at a plant density of 1.5m to 2.0 m. The control group of fruit produced in the normal season was harvested from 10 individual trees on May 2, 2021. Four batches of off-season fruit were produced in autumn on trees also planted in the open field and successively treated with paclobutrazol (3%) and monocyandiamide (1.5%). The off-season fruit were harvested on September 21, September 27, October 8, and October 15, 2021. The paclobutrazol and monocyandiamide treatment times and main phenological stages of the tested mulberry trees are presented in **Supplemental Table 1**.

**Figure 1:**
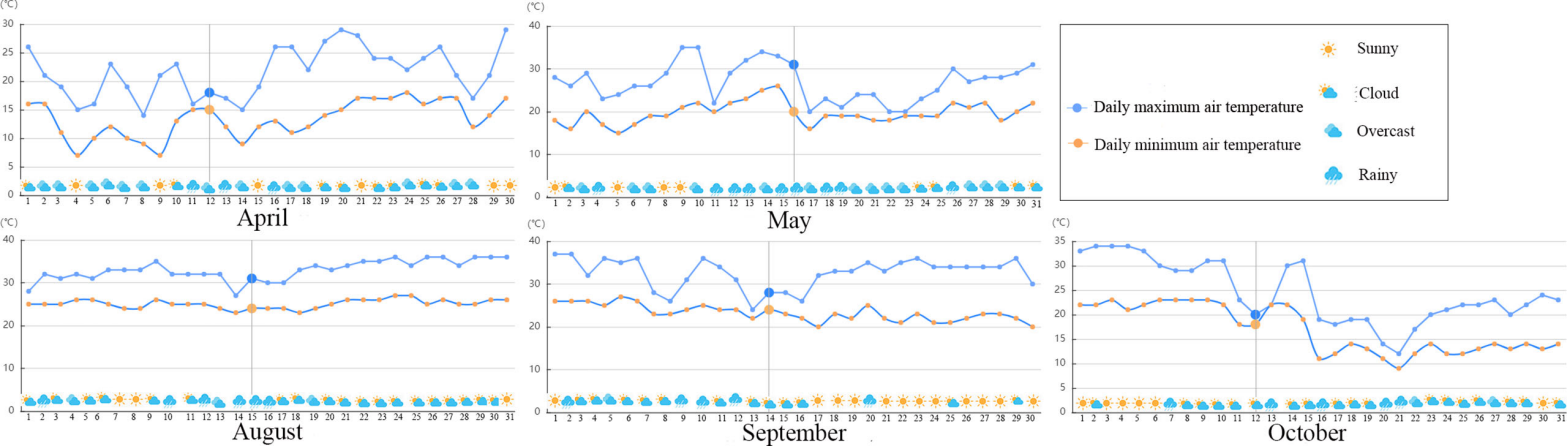
Weather conditions of mulberry cultivation base in differential months.

Meteorological data for the study area in the experimental period (**Figure 1**) were obtained from a weather network (https://www.tianqi.com/).

### Determination of agronomic characters

The fruit length, fruit diameter, and fruit stem length were measured using a Vernier caliper (Meinaite, Shanghai, China). The individual fruit weight was determined using a Mettler ML 240 electronic balance (Mettler Toledo, Greifensee, Switzerland). The pH was measured using a FiveEasy Plus pH meter (Mettler Toledo Group, Schwerzenbach, Switzerland). Soluble solids content (SSC) was measured with a refractometer (LH-B55, Lohand Biological, Hangzhou, China). Measurements were recorded for 10 replicate fruit for each group.

Moisture content was determined using the method of AOAC (2005). Ten fresh fruits from each group were accurately weighed. The fruit samples were then dried at 105 ± 1°C for 4 h, covered with a bottle cap and move to a dryer, cooled for 30 min, accurately weighed, then dried at 105°C for 1 h, cooled in the dryer, and weighed again. A difference between two successive weighings of 5 mg or less is considered to represent the constant weight. The moisture content of the fruit was calculated with the following formula:

Moisture content (%) = (fresh weight − dry weight)/fresh weight × 100.

Juice yield (%) was calculated in accordance with the method of Wen et al. (2015) with an efficiency modification. Ten fresh fruits were thoroughly ground in a mortar with a pestle after accurate weighing and the homogenate was transferred to a 50 ml centrifuge tube. After centrifugation at 10,000 *g* for 20 min, the supernatant was discarded and the weight of the pulpy residue was weighed. The juice yield was calculated with the following formula:

Juice yield (%) = (fresh fruit weight − pulpy residue weight)/fresh fruit weight × 100.

Three replicate determinations of moisture content and juice yield were performed for each group of fruit.

### Determination of nutritional components content

The crude protein content was measured using the Kjeldahl method in accordance with the official method 979.09 of the AOAC (2005). The sample powder (2 g) was mixed with 30 mL concentrated sulfuric acid in a Tecator tube. After addition of two tablets of 1000 Kjeltabs Cu/3.5 catalyst mixture to the tube, the mixture was allowed to digest using a Foss digester for 1 h at 420°C. A clear solution was obtained after approximately 1 h. The distillation and titration processes were conducted using a Foss Kjeltec 8400 analyzer (Foss Analytical, Hoganas, Sweden) with a receiver solution of 40% NaOH and 0.1 N HCl as titrant solutions and deionized water. Recovery was checked using 0.3 g ammonium iron (II) sulphate hexahydrate with theoretical nitrogen values of ammonium sulphate (21.09%) during this analysis. The percentage crude protein was calculated using the conversion factor of 6.25 in triplicate.

The total sugar content was determined using the phenol–sulfuric acid method. The freeze-dried fruit powder (0.25 g) was dissolved in 10 mL distilled water, boiled in a boiling water bath for 30 min, and then centrifuged at 8000 g for 5 min. The same sample was extracted twice. A portion of the merged supernatant solution (2.0 mL) was diluted to 80 mL. Next, 1.0 mL of the diluted solution was mixed with 1.0 mL of 6% phenol and shaken, then 5.0 mL concentrated H_2_SO_4_ was added and allowed to settle for 10 min. The solution was cooled with water to the ambient temperature and the absorbance was measured with an Infinite M200 spectrophotometer (Tecan, Seestrasse, Switzerland) at a wavelength of 485 nm. The sugar content was determined from a standard curve for glucose.

The reducing sugar content was determined with the 3,5-dinitrosalicylic acid (DNS) colorimetric method. The freeze-dried fruit powder (0.2 g) was dissolved in 8 mL distilled water and heated at 100°C in a water bath for 30 min. The supernatant of the reaction mixture was obtained by centrifugation at 8000 g for 5 min. A portion of the supernatant (500 µL) was diluted 40 times with distilled water. Next, 1 mL of the diluted supernatant was mixed with 1 mL DNS solution and decocted for 5 min in boiling water at 100°C. After cooling to ambient temperature, the absorbance was measured at on an Infinite M200 spectrophotometer (Tecan, Seestrasse, Switzerland)) at a wavelength of 540 nm. The reducing sugar content was determined from a standard curve for glucose.

The content of total flavonoids was measured with the sodium nitrite–aluminum nitrate–sodium hydroxide colorimetric method. Approximately 0.1 g of freeze-dried fruit powder was extracted with 1 mL of 80% ethanol, ultrasonicated for 30 min at room temperature, and then centrifuged at 8000 g for 10 min. A portion of the supernatant (500 µL) was diluted with 4.5 mL of 80% ethanol. After dilution, 6 mL diluted supernatant was mixed with 1 mL of 5% NaNO_2_ and incubated for 6 min. The mixture was added to 1 mL of 10% Al(NO_3_)_3_ and incubated for 6 min. The mixture was added to 10 mL of 4% NaOH solution and the volume was made up to 25 mL with 80% ethanol. After incubation for 15 min, the absorbance was determined at 500 nm and measured relative to a blank extraction solvent. The total flavonoid content was expressed as rutin equivalents per gram of dry sample.

Total polyphenol content was determined in accordance with the Folin–Ciocalteu method with minor modifications. Approximately 0.1 g freeze-dried fruit powder was extracted with 1 mL of 95% ethanol for 1 h in a water bath at 65°C and then centrifuged at 8000 g for 10 min. A portion of the supernatant (0.2 mL) was diluted with 4 mL distilled water and 1.8 mL of 20% aqueous Na_2_CO_3_ solution was added. Next, 0.4 mL Folin–Ciocalteu reagent was added, and the mixture was incubated in the dark at room temperature for 2 h. Finally, the absorbance was measured with a spectrophotometer at 765 nm (Infinite M200, Tecan, Seestrasse, Switzerland). Total polyphenol content was expressed as gallic acid equivalents per gram of dry sample.

The total acid content was determined using the potentiometric titration method with slight modifications. Approximately 0.1 g freeze-dried fruit powder was extracted with 1 mL of ethanol containing 1% (v/v) formic acid and then sonicated for 30 min. After calibration, the pH electrode was immersed in the test solution, then the test solution was titrated using 0.1 mol/L NaOH standard titration solution. The NaOH standard titration solution was added dropwise until pH 8.2 was attained. The volume of NaOH standard titration solution added to the test solution was recorded. The total acid content was estimated using the following formula:

Total acid content (mg/g) = [*C* × (*V*_1_ – *V*_2_) × 0.064]/*m* × 1000

where *C* is the concentration of NaOH standard titration solution (mol/L), *V*_1_ is the volume of NaOH standard solution consumed by the sample dilution after titrating the sample dilution with formaldehyde to the end point (pH 8.2), *V*_2_ is the volume of the NaOH standard solution consumed by the blank test after titration with formaldehyde to the end point (pH 8.2), 0.064 is the conversion factor for citric acid, and *m* is the mass of fruit powder for analysis (g).

The anthocyanin content was determined using the pH differential method. Approximately 0.1 g sample powder was extracted with 1 mL of 80% ethanol containing 1% (v/v) HCl and then sonicated for 30 min at room temperature in the dark. After centrifugation at 8000 g for 10 min, 0.5 mL of the supernatant was diluted with 4.5 mL of 80% ethanol containing 1% (v/v) HCl. The absorbance of the diluted supernatant was measured at 530 and 657 nm using a spectrophotometer (Infinite M200, Tecan, Seestrasse, Switzerland). The anthocyanin content was calculated using the following formula:


$$Anthocyanin\;content\;=\;\left(A_{530}–\;1/4\;\times A_{657}\right)\;\times N\times M/(\epsilon \times m)$$

where *N* is the dilution factor, *M* is molecular weight of cyanidin-3-glucoside (449.2), and *ϵ* is the molar absorptivity of cyanidin-3-glucoside (26,900 M^−1^ cm^−1^), and *m* is the mass of fruit powder used for extraction (g).

The content of vitamin C was evaluated using a high-performance liquid chromatograph (HPLC). A fresh fruit sample (1 g) was added to 5 mL of 10% metaphosphoric acid and vortexed for 3 min. After incubation at 4°C for 1 h, the sample was centrifuged at 8000 g for 5 min at 4°C. The vitamin C in the supernatant was determined using a Waters 600 HPLC (Waters Corp., Milford, MA, USA) equipped with a LiChrospher C18 column (250 × 4.0 mm, 5 µm) fitted with the same guard column. The gradient of mobile phase consisted of methanol (A) and 5 mM KH_2_PO_4_ (B). A gradient mode was applied at the flow rate of 0.8 mL/min, starting with 5% A to 22% A in 6 min and returning to the initial conditions within the following 9 min. The injection volume was 20 µL, and the temperatures in the automatic injector and column oven were maintained at 15°C and 26°C, respectively. Detection was made at 254 nm by using a Waters 916 photodiode-array detector.

### Determination of sugar contents

The freeze-dried fruit powder (0.2 g) was added to 4 mL of 80% ethanol and extracted by ultrasonication for 1 h, and then incubated in a 65°C water bath for 20 min. After cooling naturally to room temperature, the extract was centrifuged at 8000 g for 10 min and supernatant was collected. The precipitate was extracted twice with same process. The collected supernatants were combined and evaporated in a water bath at 70°C. The volume was adjusted to 1 mL with distilled water. The soluble sugar extract supernatant was filtered using a 0.45 μM microporous filter membrane.

Separation of the filtered supernatant was performed with a Sugar-PaK I column (4.6 mm × 250 mm × 5 μm)under a detection wavelength of 210 nm and flow rate of 0.5 mL/min with the mobile phase (deionized, bacteria-free water containing 0.0001 M calcium EDTA). The column temperature was 30°C and the injection volume was 20 μL. This method was repeated three times for each sample.

### Validation and expression analysis of anthocyanin biosynthesis genes

Seven genes associated with anthocyanin biosynthesis were selected for quantitative real-time PCR (qPCR) assays. First-strand cDNA was synthesized using a FastQuant RT Kit (TIANGEN Biotech, KR106, Beijing, China). Gene-specific primers for qPCR were designed using R version 3.1.3 (http://cran.r-project.org/), and all primers are listed in **Supplemental Table 2**. The β-actin gene was used as an internal control to normalize gene expression. The qPCR assays were performed using SYBR Premix Ex Taq II (Tli RNaseH Plus) (Takara Bio, Shiga, Japan) in a LightCycler 480 instrument (Roche Diagnostics, Mannheim, Germany). The fold changes in expression were calculated with the 2^–ΔΔCt^ method (Livak and Schmittgen, 2001). To ensure reproducibility and reliability, three biological replicates were analyzed for each gene.

### Determination of total antioxidant activity and DPPH radical scavenging activity

The total antioxidant capacity was measured using a colorimetric method with the Total Anti-Oxidative Capability Assay Kit (A015, Nanjing Jiancheng Bioengineering Institute, Nanjing, China). The freeze-dried fruit powder was mechanically homogenized in 0.9% w/v saline solution with a mixture ratio of 1: 9 (w/v) on ice. The homogenate was centrifuged at 8000 g for 10 min at 4°C. The buffer solution, ABT solution, peroxide solution, Trolox solution, and samples were added and mixed thoroughly followed the manufacturer’s instructions. After standing for 10 min at room temperature, the optical density of each tube was measured at 520 nm with a spectrophotometer (Infinite M200, Tecan, Seestrasse, Switzerland).

The DPPH radical scavenging activity was measured with a DPPH assay kit (A153-1-1, Nanjing Jiancheng Bioengineering Institute). The freeze-dried fruit powders (5 g) were homogenized in 45 mL methanol (80%, v/v) for 30 min and then centrifuged at 8000 g for 5 min at 4°C. A portion of the supernatant (0.2 mL) was mixed with 5.8 mL of 0.1 mM DPPH in methanol and shaken vigorously. The mixture was allowed to stand at 25°C for 30 min in the dark. The absorbance was determined at 517 nm with a spectrophotometer (Infinite M200, Tecan, Seestrasse, Switzerland). The control samples without the fruit extract supernatant contained all chemical reagents mentioned above.

The total antioxidant activity and DPPH scavenging activity were respectively calculated using the equation provided with the assay kit.

### Statistical analysis

All data were expressed as the mean ± standard deviation (SD) of all replications. The significance of differences between means was analyzed using SPSS 23.0 statistical software (IBM Corp., Armonk, NY, USA). The means were considered to be statistically significant at the 5% significance level.

## Results

### Fruit agronomic characters

**Figure 2:**
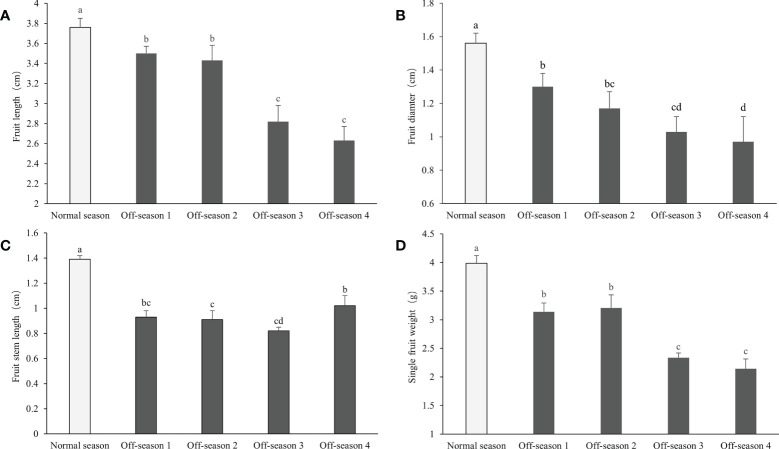
Fruit length, fruit diameter, fruit stem length, and single fruit weight of differential groups’ mulberry fruits. **(A)** Fruit length, **(B)** fruit diameter, **(C)** fruit stem length, **(D)** single fruit weight. Within a panel, bars labeled with different lowercase letters differ significantly (*P<* 0.05).

**Figure 3:**
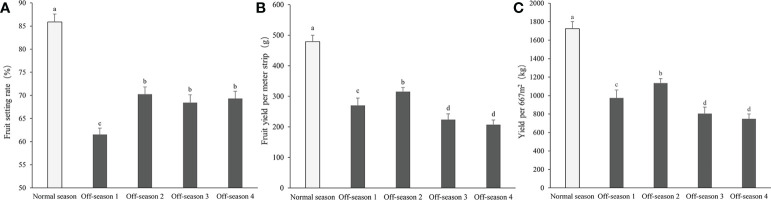
Fruit setting rate, fruit yield per meter strip, and yield per 667m^2^ of differential groups’ mulberry fruits. **(A)** Fruit setting rate, **(B)** fruit yield per meter strip, **(C)** fruit yield per 667m^2^. Within a panel, bars labeled with different lowercase letters differ significantly (*P<* 0.05).

The morphological appearance of differential groups’ mulberry fruits was given in **Supplemental Figure 1**. After statistical measurement, the fruit length, fruit diameter, fruit stem length, and single fruit weight of the different batches of off-season fruit were found to be all significantly lower than those of fruit produced in the normal season (*P<* 0.05; **Figure 2**). Thus, the off-season fruit were notably smaller than the normal-season fruit. In addition, the percentage fruit set, fruit yield per meter strip, and fruit yield per 667 m^2^ of the off-season fruit were significantly lower than those of the normal-season fruit (**Figure 3**). The small size and lower yields of off-season fruit may be associated with the dry weather in autumn and short growth cycle.

**Figure 4:**
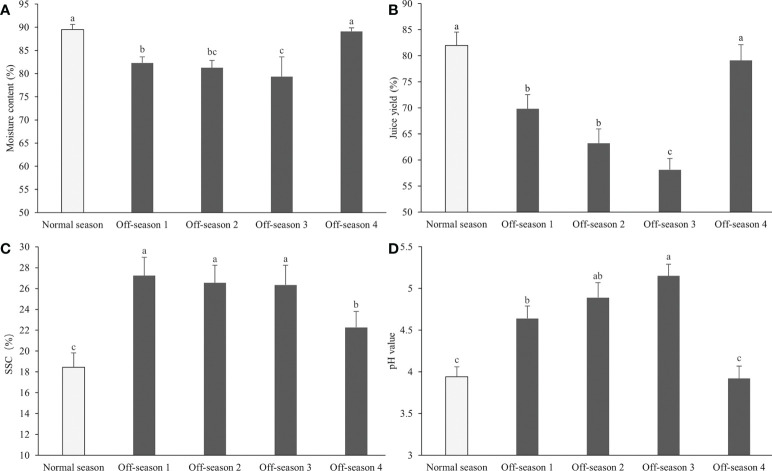
Moisture content, juice yield, pH value and SSC content of differential groups’ mulberry fruits. **(A)** Moisture content, **(B)** juice yield, **(C)** SSC, **(D)** pH value. Within a panel, bars labeled with different lowercase letters differ significantly (*P<* 0.05).

The taste of the normal-season and off-season 4 fruit was slightly more sour than that of the other three off-season fruit batches. Significant differences were observed in agronomic characters among the fruit groups, such as moisture content, juice yield and pH, and SSC (**Figure 4**). The moisture content and juice yield of off-season fruit, except for the off-season 4 fruit, were significantly lower than those of normal-season fruit (*P<* 0.05; **Figures 4A, B**). The SSC of the fruit juice were significantly higher in all off-season fruit groups than those of the normal-season fruit (*P<* 0.05; **Figure 4C**), and the fruit juice pH of off-season 1, 2 and 3 were significantly higher in all off-season fruit groups than those of the normal-season fruit (*P<* 0.05; **Figure 4D**).

### Nutritional composition

**Figure 5:**
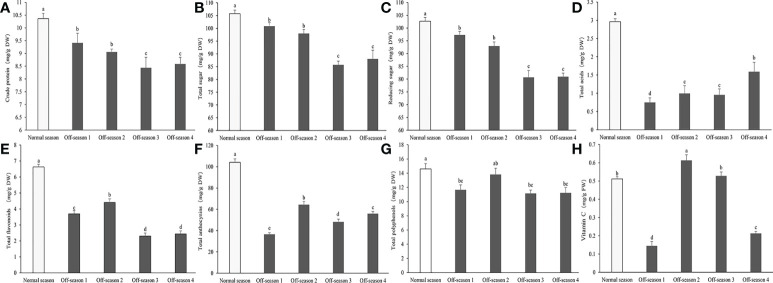
Contents of main nutritional composition in differential groups’ mulberry fruits. **(A)** Crude protein, **(B)** total sugar, **(C)** reducing sugar, **(D)** total acids, **(E)** total flavonoids, **(F)** total anthocyanins, **(G)** total polyphenols, **(H)** vitamin (C) Within a panel, bars labeled with different lowercase letters differ significantly (*P<* 0.05).

The contents in the mass fraction of crude protein, total sugars, reducing sugars, total acids, total anthocyanins, and total flavonoids were decreased significantly in all batches of off-season fruit compared with those of the normal-season fruit (*P<* 0.05; **Figure 5**). The total acid content of the normal-season and off-season 4 fruit were significantly higher than other of the three batches of off-season fruits (**Figure 5D**). This was an important in the slightly sour taste of the normal-season and off-season 4 fruit. Compared with normal-season fruit, the total polyphenol content was decreased significantly in off-season 1, 3, and 4 fruit (*P<* 0.05), but not in off-season 2 fruit (**Figure 5F**). The vitamin C content was significantly lower only in off-season 1 and 4 fruit compared with that of the normal-season fruit (*P<* 0.05; **Figure 5H**).

### Sugar contents

**Figure 6:**
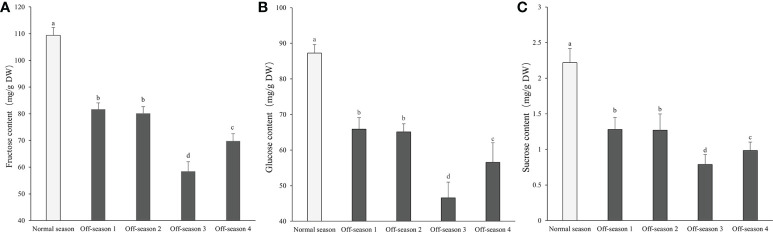
Contents of fructose, glucose, and sucrose in differential groups’ mulberry fruits. **(A)** Fructose content, **(B)** glucose content, **(C)** sucrose content. Within a panel, bars labeled with different lowercase letters differ significantly (*P<* 0.05).

The fructose content of the mulberry fruit was 49.27–74.12 times higher than the sucrose content, and the glucose content was 39.32–59.12 times higher than the sucrose content (**Figure 6**). The fructose, glucose, and sucrose contents of the off-season fruit were all notably lower than those of the normal-season fruit (*P<* 0.05). The contents of fructose, glucose, and sucrose of the off-season 3 and 4 fruit were significantly lower than those of the off-season 1 and 2 fruit (*P<* 0.05). The fructose, glucose, and sucrose contents of the off-season 3 fruit were significantly lower than those of all other groups of fruit (*P<* 0.05).

### Anthocyanin biosynthesis structural gene expression

**Figure 7:**
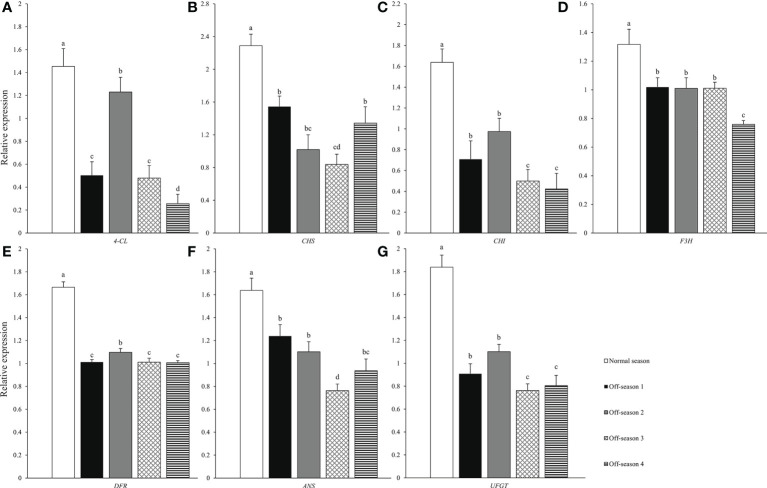
Anthocyanin synthesis structure genes between differential groups’ mulberry fruits. **(A)** *4-CL*, **(B)** *CHS*, **(C)** *CHI*, **(D)** *F3H*, **(E)** *DFR*, **(F)** *ANS*, **(G)** *UFGT*. Within a compound, bars labeled with different lowercase letters differ significantly (*P<* 0.05).

The expression levels of seven structural genes related to anthocyanin biosynthesis pathway among the different fruit groups were analyzed using qPCR (**Figure 7**). The expression levels of seven structural genes related to anthocyanin biosynthesis of the off-season fruit were all significantly lower compared with those of the normal-season fruit (*P<* 0.05). The expression levels of the seven genes in the fruit varied among the four off-spring batches. The anthocyanin content of the different groups of fruit (**Figure 5F**) was consistent with the expression levels of the anthocyanin synthesis structural genes.

### Total antioxidant activity and DPPH radical scavenging activity

**Figure 8:**
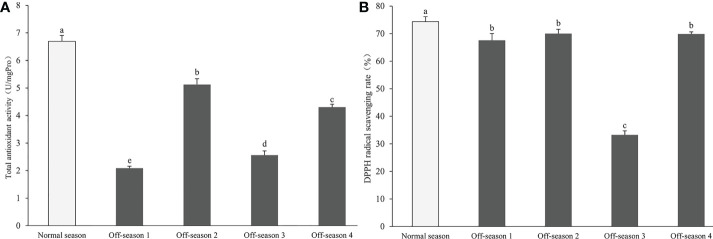
Total antioxidant activity and DPPH radical scavenging activity in differential groups’ mulberry fruits. **(A)** Total antioxidant activity, **(B)** DPPH radical scavenging rate. Within a panel, bars labeled with different lowercase letters differ significantly (*P<* 0.05).

The total antioxidant activity in all off-season fruit groups was significantly lower than that of the normal-season fruit (*P<* 0.05). In addition, the activity varied markedly among the batches of off-season fruit (*P<* 0.05; **Figure 8A**). The DPPH radical scavenging activity of off-season fruit was notably lower than that of the normal-season fruit (*P<* 0.05). Furthermore, the activity in off-season 3 fruit was significantly lower than that of the other three batches of off-season fruit (*P<* 0.05; **Figure 8B**). The total antioxidant activity and DPPH radical scavenging activity of off-season 3 fruit were significantly lower than those of all other groups of fruit (*P<* 0.05).

## Discussion

Off-season cultivation techniques have been used to produce high-value horticultural products, such as vegetables, fruits, flowers, and landscaping trees, to obtain higher returns and to meet market demand, and such techniques have been expanded considerably in recent years (Kumar et al., 2020). Application of off-season cultivation techniques for mulberry would enable regulation of the mulberry fruiting time to enhance fruit production with the aim of achieving a high market value. In the present study, the yield and quality of off-season mulberry fruit cultivated in an open field was analyzed to explore the practical application of off-season mulberry fruit production.

Fruit yield and quality are strongly linked to environmental variables of the orchard, such as soil water content, air and canopy temperature, light intensity, and wind speed (Ali et al., 2021; Sellitto et al., 2021). In the present study, the moisture content and juice yield of mulberry fruit produced in the off-season were lower than those of normal-season fruit, whereas the juice pH and fruit SSC were higher. Similar results have been observed in guava fruit, in which the total soluble solids (TSS) content of off-season (winter) fruit was higher than of main-season (rainy season) fruit (Singh et al., 1996; Mamun et al., 2008; Ekram et al., 2019). Dissimilar results were reported by Punthi and Jomduang (2021), who observed that the moisture and TSS contents in off-season mulberry fruit were significantly lower than those of normal-season fruit. The SSC is an important variable for determining the sweetness, maturity, and market value of fruit (Tian et al., 2018).

The fruit shape (length and diameter) and fruit weight of off-season mulberry fruit were lower than those of normal-season mulberry fruit. The low fruit moisture content may be an important factor influencing the fruit shape and weight. In addition, the lower percentage fruit set, smaller shape, and lower individual fruit weight influence the yields of off-season mulberry fruit. Similar results were reported in previous studies of off-season fruit, such as mango (Prasad et al., 2015; Anusuya et al., 2018), longan (Yang et al., 2010), guava (Ekram et al., 2019), and salak (Rai et al., 2014). In mango, the length of the panicle, number of panicles per square meter, and fruit yield per plant of off-season fruit were significantly lower than those of main-season fruit (Prasad et al., 2015; Anusuya et al., 2018). Yang et al. (2010) reported that small fruit size, severe fruit drop, and heavy fruit cracking were significant in off-season (winter) fruit than in-season fruit of longan. In guava, individual fruit weight was significantly higher in off-season fruit than in-season fruit, regardless of the cultivar or fruit-thinning method (Ekram et al., 2019). In addition, the yield per tree of off-season fruit was decreased compared with main-season fruit of guava (Mohsen, 2018). With regard to Salak Gula Pasir, the weight per fruit, number of fruits per plant, and weight of fruit per plant in the off-season (Ga du season) were significantly lower than those of fruit in the normal season (Sela season II) (Rai et al., 2014). Consistent with these reports, the differences in mulberry fruit yield and quality may reflect environmental differences between the in-season (spring) and off-season (autumn), particularly in temperature, illumination, and humidity.

Numerous studies have revealed that the nutritional composition of mulberry fruit is beneficial for human health, especially flavonoids, anthocyanins, vitamins, polyphenols, and sugars (Zhang et al., 2018; Bhattacharjya et al., 2021). Compared with the normal-season mulberry fruit, the contents of crude protein, total flavonoids, total sugars, reduced sugars, total acids, anthocyanins, and total polyphenols were significantly decreased in off-season fruit in this study. Previous studies have revealed that the contents of polyphenols, anthocyanins, and flavonoids in mulberry fruit produced in an open field (maturing at the end of May) were higher than those produced in a greenhouse (maturing in early May) (Lee et al., 2015a; Lee et al., 2015b). In addition, a similar result was reported by Shahab et al. (2020), who observed that total acid and total polyphenol contents tended to be lower in off-season fruit of table grape compared with main-season fruit. In contrast to these results, the major chemical constituents of off-season fruit of mango were higher compared with those of main-season fruit, such as total sugars, reducing sugars, non-reducing sugars, starch, TSS, ascorbic acid, titratable acidity, phenols, flavonoids, carotenoids, and lycopene (Prasad et al., 2015).

Anthocyanins are bioactive water-soluble plant pigments and are potentially beneficial to human health (Verediano et al., 2021). Mulberry fruit are rich in anthocyanins and have been used as a traditional medicine and functional food (Kim et al., 2020; Bhattacharjya et al., 2021). Numerous studies have indicated that 4-coumarate coenzyme A ligase gene (*4-CL*), chalconesynthase gene (*CHS*), chalconeisomerase gene (*CHI*), flavanone 3-hydroxylase gene (*F3H*), dihydroflavonol 4-reductase gene (*DFR*), anthocyanidin synthase gene (*ANS*), and UDP-glycose flavonoid glycosyltransferase gene (*UFGT*) are the important structural genes related to the anthocyanin biosynthesis pathway of plants (Li et al., 2020). In the current study, the anthocyanin content and expression levels of anthocyanin biosynthesis-related genes of off-season fruit were significantly lower than those of normal-season fruit. Similar findings were obtained by Punthi and Jomduang (2021), who reported that the anthocyanin content of off-season mulberry fruit produced in Thailand in August (off-season) was significantly lower than that of fruit produced in February (in-season); however, no distinct differences in anthocyanin content was observed between off-season mulberry fruit produced in November and in-season fruit. A sweet taste is an important quality attribute for fruit and is usually associated with the sucrose, glucose, and fructose contents of the fruit (Gomez et al., 2002). The contents of glucose, fructose, and sucrose were significantly lower in the off-season mulberry fruit than in the normal-season fruit. Solar radiation and temperature have been indicated to have a strong influence on fruit sugar accumulation (Beckles, 2012; Dong et al., 2019). In the present study, the decrease in the main nutritional components of off-season mulberry fruit may be associated with the short-day length and the effect of photosynthesis and sugar accumulation.

Several previous studies have reported that mulberry fruit exhibit favorable antioxidant potential, and the fruit have long been used for their antioxidant and antibacterial properties (Bhattacharjya et al., 2021; Li et al., 2021). In the present study, the total antioxidant activity and DPPH activity of the off-season fruit were significantly lower than those of the normal-season fruit. Consistent with this finding, Punthi and Jomduang (2021) observed that antioxidant activities measured in DPPH and TRAP assays were higher in in-season mulberry fruit than in off-season fruit. The antioxidant activity, measured on the basis of DPPH free radical scavenging effects and FRAP, is higher in tomato fruit produced in an open field than in a greenhouse (Patanè et al., 2019). The decreased antioxidant potential of off-season mulberry fruit may be preliminarily attributed to their low contents of antioxidant compounds, such as flavonoids, anthocyanins, and polyphenols.

## Conclusion

In summary, this study shows that the yield and quality of off-season mulberry fruit are inferior to mulberry fruit produced in the normal season. This may be due to the unfavorable climate in autumn and breaking dormancy by treatment with paclobutrazol and monocyandiamide. With the aim of achieving a high market value using off-season techniques, we hope to improve the fruit quality and yield by adjusting environmental factors and selection of a suitable alternative to paclobutrazol and monocyandiamide to break dormancy.

## Data availability statement

The raw data supporting the conclusions of this article will be made available by the authors, without undue reservation.

## Author contributions

JW, LC designed the study. PL, YZ, LX, and ZX performed the experiments. PL, JY, and ZL analyzed the data and wrote the manuscript. ZX and TL collected and prepared the samples. All authors contributed to the article and approved the submitted version.

## Funding

This work was supported by the Earmarked Fund for Key Scientific and Technological Grant of Zhejiang for Breeding New Agricultural Varieties (2021C02072-5), Major Agricultural Technology Collaborative Extension Project of Zhejiang Province (2022XTTGCS01), Public Welfare Technology Plication Research Project of Zhejiang Province of China(LGN21C160013), Zhejiang Provincial Natural Science Foundation of China (LZ21C020001), and the Modern Agro-industry Technology Research System of China (CARS-18-ZJ0203).

## Conflict of interest

The authors declare that the research was conducted in the absence of any commercial or financial relationships that could be construed as a potential conflict of interest.

## Publisher’s note

All claims expressed in this article are solely those of the authors and do not necessarily represent those of their affiliated organizations, or those of the publisher, the editors and the reviewers. Any product that may be evaluated in this article, or claim that may be made by its manufacturer, is not guaranteed or endorsed by the publisher.
