# Supplementary figures and images for: Biological characteristics, bioactive compounds, and antioxidant activities of off-season mulberry fruit
Source: Front Plant Sci. 2022 Oct 25;13:1034013. doi: 10.3389/fpls.2022.1034013 (PMC9667739; doi:10.3389/fpls.2022.1034013)

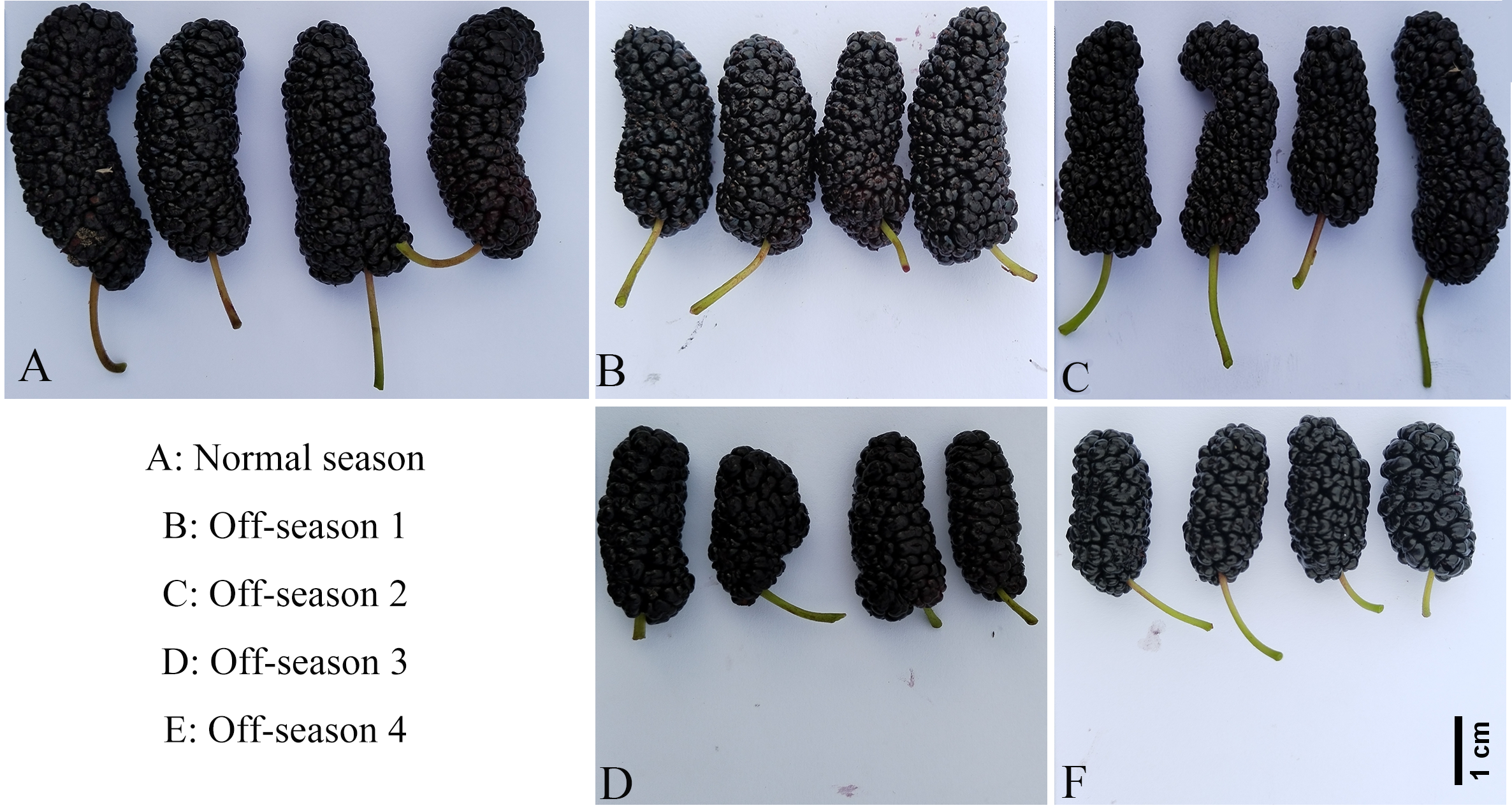

Supplement: Supplementary file 1 [file Image_1.tif]
