# Supplementary material for: Biological characteristics, bioactive compounds, and antioxidant activities of off-season mulberry fruit
Source: Front Plant Sci. 2022 Oct 25;13:1034013. doi: 10.3389/fpls.2022.1034013 (PMC9667739; doi:10.3389/fpls.2022.1034013)
Supplement: Supplementary file 2 [file Table_1.docx]

**Table 1. Treat time of paclobutrazol and monocyandiamide and main phenophase of mulberry**

| Batch | Treat time | | Phenophase | | |
| --- | --- | --- | --- | --- | --- |
|  | Paclobutrazol | Monocyandiamide | Germination time | Time of fruit star ripening | Time of most of fruits ripening |
| CK | — | — | February 27 | April 25 | May 2 |
| OSM-1 | July 12 | August 3 | August 11 | September 15 | September 21 |
| OSM-2 | July 12 | August 9 | August 19 | September 20 | September 27 |
| OSM-3 | July 12 | August 17 | August 26 | September 25 | October 1 |
| OSM-4 | July 12 | September 5 | September 13 | October 10 | October 15 |
