# Supplementary material for: Biological characteristics, bioactive compounds, and antioxidant activities of off-season mulberry fruit
Source: Front Plant Sci. 2022 Oct 25;13:1034013. doi: 10.3389/fpls.2022.1034013 (PMC9667739; doi:10.3389/fpls.2022.1034013)
Supplement: Supplementary file 3 [file Table_2.docx]

**Table 2 Primers for qRT-PCR analysis**

| Primer name | Forward primer | Reverse primer |
| --- | --- | --- |
| *4CL* | 5'-CACCGGCAAGACCTACACTT-3' | 5'-GGAGGAGGATCATGCAGACG-3' |
| *CHS* | 5'-CCTCCTCAAGGATGTTCCCG-3' | 5'-ACTTGGTCGAGAATGGCTGG-3' |
| *CHI* | 5'-AACCGGCCCATTTGAAGT-3' | 5'-TTGCCACGCAATTTTCCGAC-3' |
| *ANS* | 5’-GCTTGCGACCACCATACTTT-3’ | 5’-TGCCGTCACCCATTTGC-3’ |
| *DFR* | 5’-TTGTCGGACCGTAAAGATG-3’ | 5’-GCAGTGGGACCAAGAAAT-3’ |
| *F3H* | 5’-CGCCGTGCTGAGATTTG-3’ | 5’-CTAACCGCTTCACCCTGTA-3’ |
| *UFGT* | 5’-GAGACGGGGAGGGAGGTTAG-3’ | 5’-GGGTTCGTTTTCACTTCCTTTAG-3’ |
| *β-actin* | 5'-AAGTCATCACAATCGGAG-3' | 5'-GGGAACATAGTTGAACCA-3' |
